# Key Amino Acids for *Pepper Vein Yellows Virus* P0 Protein Pathogenicity, Gene Silencing, and Subcellular Localization

**DOI:** 10.3389/fmicb.2021.680658

**Published:** 2021-09-13

**Authors:** Lishuang Wang, Peijie Tian, Xiuling Yang, Xueping Zhou, Songbai Zhang, Chun Li, Xuehui Yang, Yong Liu

**Affiliations:** ^1^College of Plant Protection, Hunan Agricultural University, Changsha, China; ^2^Institute of Plant Protection, Guizhou Academy of Agricultural Sciences, Guiyang, China; ^3^State Key Laboratory for Biology of Plant Diseases and Insect Pests, Institute of Plant Protection, Chinese Academy of Agricultural Sciences (CAAS), Beijing, China; ^4^Institute of Plant Protection, Hunan Academy of Agricultural Sciences, Changsha, China

**Keywords:** *pepper vein yellows virus*, *Polerovirus*, pathogenic factor, RNA silencing suppressor, subcellular localization

## Abstract

*Pepper vein yellows virus* (PeVYV) is a newly recognized *Polerovirus* extracted from Chinese pepper. The symptoms of PeVYV-infested pepper plants comprise intervein yellow staining, leaf curl formation and other malformations, and leaf internodal shrinkage, but the roles of the viral proteins remain undetermined. The P0 protein of the genus *Polerovirus* has established post-transcriptional gene silencing (PTGS) activity. This investigation focused on the PeVYV-encoded P0 protein and assessed its potential virulence capacity, PTGS activity, and tendencies to localize in the nucleus. This study revealed that P0 influenced the pathogenic properties of a specific heterologous *potato virus X*. In addition, P0 proteins impaired local gene silencing, although they did not regulate generalized gene silencing within *Nicotiana benthamiana* 16c plants. Furthermore, P0 proteins localized mainly in the nucleus, particularly in the nucleolus. P0 deletion mutagenesis demonstrated that the F-box motif (56–72 amino acids, AAs) of P0 was essential for symptom determination, inhibition of PTGS, and subcellular localization. Mutation analysis of the F-box motif of P0 protein indicated that AA 57 of the P0 protein was a pivotal site in symptom development and that AA 56 of the P0 protein was indispensable for inhibiting PTGS and subcellular localization. The outcomes obtained here suggest that further studies should be conducted on the molecular mechanisms of amino acids of the F-box domain of P0 protein in the interaction of PeVYV with plants.

## Introduction

Pepper (*Capsicum* spp.) is a major agricultural crop grown in China, but one of its main biotic threats is *pepper vein yellow virus* (PeVYV), which is a notorious pathogen due to its geographical distribution and its linkage to severe agricultural crop yield losses. PeVYV was initially identified in Japan in 1995 and is transmitted by the *Aphis gossypii* Glover aphid (Yonaha et al., 1995). The symptoms of PeVYV comprise intervein yellow staining, leaf curl formation and other malformations, leaf puckering, and internodal shrinking. Such symptom traits have also been identified in pepper, tomato, tobacco, black nightshade, and other *solanaceous* plants from a spectrum of regions within Africa, Asia, and Europe (Dombrovsky et al., 2010; Murakami et al., 2011; Knierim et al., 2013; Villanueva et al., 2013; Alfaro et al., 2014; Alabi et al., 2015; Tan et al., 2015; Zhang et al., 2015; Liu et al., 2016; Afouda et al., 2017; Wang et al., 2017).

*Pepper vein yellow virus* belongs to the family Luteoviridae of the genus *Polerovirus*, whose genomes consist of single linear, positive-sense, single-stranded RNA containing 6,244 nucleotides (nt), including six open reading frames (ORFs; ORF0 to ORF5) (Liu et al., 2016). ORF0 expresses an RNA-silencing suppressor protein, P0, whereas ORF1 and ORF2 express viral replication proteins such as viral RNA polymerase. The main coat protein (CP) is expressed from ORF3 (3’ section). The ORF4 region, which is present within the CP gene, although on an alternative reading frame, leads to the expression of the viral movement protein. ORF5 can be expressed through casual CP termination codon suppression and also encodes the read-through domain, which is necessary for effective aphid transmission (Ali et al., 2018; Agrofoglio et al., 2019; Avelar et al., 2020).

Throughout the past few years, P0 proteins derived from multiple *poleroviruses* have been demonstrated to act as RNA silencing suppressors (RSSs) (Almasi et al., 2015; Cascardo et al., 2015; Chen et al., 2016; Wang et al., 2018; Agrofoglio et al., 2019; Li et al., 2019). Multiple investigations have assessed the necessity for an F-box-like motif or P0–S phase kinase-associated protein 1 (SKP1) involvement in RNA silencing inhibition (Almasi et al., 2015). The polerovirus-F-box P0 protein can induce AGONAUTE 1 (AGO1) protein disintegration as a viral countermeasure. The implementation of silencing inhibitors is typically synonymous with virulence traits. Mutations of the F-box-like motif mostly occur in tandem with the loss of HR elicitations, and both of these effects lead to a reduction in *Turnip yellow virus* (TuYV) P0 viral suppressors of RNA silencing/RNA interference (VSR) activities (Wang et al., 2015). The consensus minimal F-box-like motif (LPXX(L/I) X10-13P) in P0 proteins has been postulated to be essential for RNA silencing inhibition because its mutation affects P0-based RNA silencing inhibition properties (Pazhouhandeh et al., 2006). Nevertheless, the amino acid(s) involving the function(s) of the P0 protein still remains unclear.

Through P0 protein amino acid sequence analyses using samples obtained in Guizhou Province in China, its unique F-box motif could be identified (56 LPFLLSSQCPFLSGNTP72). The purpose of this study was to elucidate how the action of P0 could also provide a strategic angle of knowledge regarding the virulence roles of this protein as well as – ultimately – *Polerovirus* pathogenesis. This investigation assessed virulence variables and RNA silencing inhibitors, all of which were derived from the PeVYV P0 protein.

## Materials and Methods

### Plasmid Design and Development

The P0 ORF was amplified from complementary DNA (cDNA) (Guizhou variant). The polymerase chain reaction (PCR) products were individually cloned into the pGEM-T Easy vector^®^ (Transgen^TM^, Beijing, China) to generate pGEM-T-P0, which served as a substrate for specific enzymes to allow cloning. The mutant P0^*dm56–72aa*^, in which the nucleotide sequences of 56–72 amino acid residues of the P0 protein were deleted, was constructed using the overlap PCR method (Bryksin and Matsumura, 2018). The mutants P0^△^^56,57aa^, P0^△^^56a^, and P0^△^^57a^, in which the amino acid(s) were replaced by arginine (A), were manufactured through the Fast Mutagenesis System^®^ (Transgen^TM^, Beijing, China) using unique primer sets for on/around mutation sites (Table 1). To test for pathogenicity, the P0 ORF, together with mutated variants, was cloned within a PVX-containing pGR106 vector (mid-sequence inside *Cla*I and *Sal*I restriction site boundaries) to yield PVX-P0.

**TABLE 1:** Synthetic oligonucleotide primers.

Primers	Sequence (5′–3′)
**Primers used for the construction of recombinant PVX vector or pCHF3-based binary vectors**	
P0-BamHI ClaI-F	***GGATCCATCGAT***ATGAACTTTGAATTGATCAACGGA
P0-SalI-R	***ACGCGTCGAC***TCACTGTAGTTCCTTCTGAATCTG
**Primers used to generate P0 mutant**	
P0^*dm56–72aa*^-F	CACCGGAACGGCAAGCGGGAACAG
P0^*dm56–72aa*^-R	GAGAGCACAAATAGAGCGAAGAAAATGG
P0^Δ56,57aa^-F	CTTCGCTCTATTTGTGCTCTCGCCGCTTTCCTTCTCA
P0^Δ56,57aa^-R	AGCGGCGAGAGCACAAATAGAGCGAAGAAAATGG
P0^Δ56a^-F	CTTCGCTCTATTTGTGCTCTCGCCCTTTTCCTTCTCA
P0^Δ56a^-R	AAGGGCGAGA GCACAAATAG AGCGAAGAAA ATGG
P0^Δ57a^ -F	CTTCGCTCTATTTGTGCTCTCTCCGCTTTCCTTCTC
P0^Δ57a^ -R	AGCGGAGAGAGCACAAATAGAGCGAAGAAAATGG
**Primers used for qPCR**	
qPVX-F	CAGGGTCAACTACCTCAACTAC
qPVX-R	GGCACGAGCTGTACTAAAGAA
GAPDH-F	GCAGTGAACGACCCATTTATCTC
GAPDH-R	AACCTTCTTGGCACCACCCT

*Bold represents the endonuclease sites.*

The end products consisting of recombinant PVX constructs were separately transformed through electroporation within *Agrobacterium tumefacien*s strain GV3101.

For post-transcriptional gene silencing (PTGS) inhibition investigations, full-length ORFs of P0 or P0^*dm56–72aa*^, P0^△^^56,57aa^, P0^△^^56a^, and P0^△^^57a^ were subcloned into the pCHF3 vector ^[30]^ between the *Bam*HI and *Sal*I sites for the creation of pCHF3- P0, P0^*dm56–72aa*^, P0^△^^56,57aa^, P0^△^^56a^, and P0^△^^57a^. All resultant constructs were separately transformed into the *A. tumefaciens* strain C58C1 through electroporation.

For subcellular localization analyses, the full-length segment of PeVYV P0, P0^*dm56–72aa*^, P0^△^^56,57aa^, P0^△^^56a^, and P0^△^^57a^ was incorporated within the *Bam*HI and *Sal*I sites of the pCHF3-N-eGFP vector ^[31]^ to prepare 35S-GFP-P0, P0^*dm56–72aa*^, P0^△^^56,57aa^, P0^△^^56a^, and P0^△^^57a^, which contain P0, P0^*dm56–72aa*^, P0^△^^56,57aa^, P0^△^^56a^, and P0^△^^57a^ viral N-terminal fusion protein attached to an enhanced green fluorescent protein (eGFP), respectively. The resultant plasmid was transformed within *A. tumefaciens* strain C58C1 through electroporation measures.

### Agroinfiltration

After incubation in Luria–Bertani broth with complementary antibiotics at 28°C overnight, all preparations were subjected to centrifugation/resuspension using infiltration buffer consisting of 10-mM 2-(N-morpholino)ethanesulfonic acid pH 5.7, 10-mM magnesium chloride, and 150-mM acetosyringone, after reaching an optical density at a wavelength of 600 nm of 0.5–1.0. After incubation at 25°C for 3 h, all suspensions were allowed to infiltrate within 4-week-old *Nicotiana benthamiana* foliage. At 36-h post-infiltration, these crops were consequently scrutinized for fluorescent properties by confocal laser scanning microscopy.

### Hydrogen Peroxide Detection

This analysis was performed with *N. benthamiana* foliage using the 3,3′-diaminobenzidine (DAB)– hydrochloric acid collection methodology, according to previously reported protocols with minimal optimizations (Balzergue et al., 2017). In brief, the leaves were detached, treated with reagents, incubated overnight, bleached using 96% alcohol in boiling-hot water for 5 min, and consequently subjected to fluorescence microscopy/photography. Such a technique was necessary for leaf discoloration and allowed consequent identification of hydrogen peroxide intensity distributions based on dark-brown sediments derived from interactions between DAB (reagent used in the study) and hydrogen peroxide (H_2_O_2_).

### Post-transcriptional Gene Silencing Assay

To analyze the efficiency of silencing inhibition, equivalent volumes of *Agrobacterium* cultures containing 35S-GFP and analyzed constructs were combined into one solution and then infiltrated within mature foliage of 4-week-old 16c (or wild-type) *N. benthamiana* plants. Concomitant 35S-GFP infiltration utilizing a construct expressing *tomato bushy stunt virus* P19 was used as a positive control, whereas an empty pCHF3 vector served as a negative control. *A. tumefaciens* cultures bearing 35S-GFP, together with pCHF3-P0 and its mutant variants, P19 construct, or the empty pCHF3 vector, were combined in equivalent fractions and allowed to infiltrate *N. benthamiana* 16c foliage. GFP fluorescence within infiltrated/non-infiltrated foliage was closely supervised with the aid of a UV lamp (Black-Ray^®^ Model B-100A, San Gabriel, CA, United States).

### RNA Extraction and Analysis

Total RNA was collected through TRIzol^®^ according to the manufacturer’s protocols (Invitrogen^TM^, Carlsbad, CA, United States). All heavily affected foliage of *N. benthamiana* crops treated with PVX or recombinant PVX constructs was collected and prepared for PeVYV P0 expression through quantitative reverse transcription PCR (RT-qPCR) measures. A 1-μg mass of total RNA was reverse transcribed into cDNA using *TransScript*^®^ II One-Step gDNA Removal and cDNA Synthesis SuperMix (Transgen^TM^, Beijing, China). Individual PeVYV P0/mutant expression levels were analyzed using PCR using primer sets (listed in Table 1). ρ*-*Values were calculated using unpaired two-tailed Student’s *t*-test (Li et al., 2019).

### Protein Extraction and Western Blotting

Protein extractions (total soluble), sodium dodecyl sulfate–polyacrylamide gel electrophoresis, and Western blotting analyses were conducted according to previously described protocols (Agrofoglio et al., 2019). PVX recognition was conducted by extracting the proteinaceous content from heavily infected (with PVX or PVX recombinant constructs) *N. benthamiana* leaves. Anti-CP monoclonal antibody (MAb) that targets PVX (developed at the Institute of Biotechnology, Zhejiang University, Hangzhou, China) was used (dilution factor = 1:8,000). GFP determination was performed by extracting proteins from infiltrated foliage of *N. benthamiana* line 16c.

All Western blotting assays were visualized using a secondary peroxidase-conjugated goat-based, anti-mouse antibody (Cell Signaling Technology^TM^, Boston, MA, United States) together with a chemiluminescence detection system (Tianneng^TM^, Shanghai, China).

### Fluorescence Analysis

To determine the subcellular localizations of GFP fusion proteins, fluorescent imaging of *N. benthamiana* afflicted with transformed Agrobacterium was conducted through Zeiss^TM^ LSM 880^®^ confocal laser scanning microscopy using presets for GFP (excitation at 488 nm/emission at 500–550 nm) and chloroplast autofluorescence (excitation at 561 nm/emission at 650–750 nm).

## Results

### PeVYV P0 Is a Pathogenicity Determinant

**FIGURE 1:**
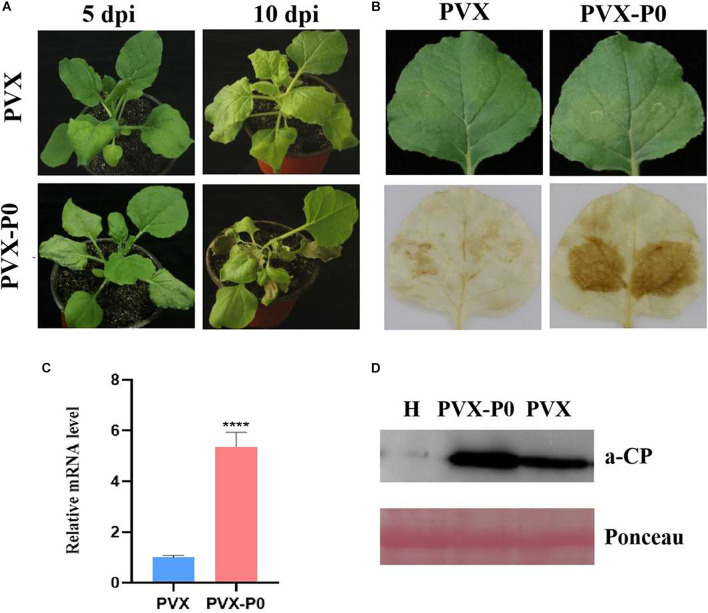
PeVYV-derived P0 protein influences potato virus X (PVX) pathogenesis. **(A)** Symptomatology of *Nicotiana benthamiana* crops infected with Agrobacterium cells carrying PVX alone or a recombinant PVX vector expressing ORF 0 of PeVYV. Images created at 5 days post-infiltration (dpi) and 10 dpi are shown. Three independent assays were conducted, and a minimum of four to six crops were utilized for each infiltration event. **(B)** DAB staining for detecting H_2_O_2_ accumulation within PVX-P0- and PVX-infected *N. benthamiana* crops at 3 dpi. Results illustrated increased accumulations of H_2_O_2_ within PVX-P0-infected foliage. **(C)** RT-qPCR analysis of PVX CP RNA accumulation within inoculated leaves at 5 dpi. Results illustrate increased accumulations of PVX CP RNA in PVX-P0-infected foliage. Values are means ± SDs. Highly significant differences (****ρ < 0.0001) between samples in each pair are indicated. **(D)** Western blotting analysis of PVX accumulation within infected *N. benthamiana* plants at 5 to 7 dpi using a monoclonal antibody targeting PVX CP. Total protein was collected from upper non-infected foliage as indicated in **(A)**. Two separate crops were used for extracting protein contents. H stands for total soluble proteins collected from *N. benthamiana* crop used for detecting PVX antibody specificity. Ponceau staining of Rubisco (large subunit) was used in loading control well.

Following the research protocols described earlier, PVX-infused leaves developed mosaic symptoms in upward leaves visible at 10 dpi, and PVX-P0-infected plants developed symptoms of wilting and necrotic lesions (Figure 1A). To validate whether the necrotic phenotype correlated with the H_2_O_2_ content, DAB reagent staining was used. As shown in Figure 1A, leaves inoculated with PVX-P0 induced H_2_O_2_ bursts, suggesting that PVX-P0 could act as a symptom inducer. RT-qPCR was used to investigate PVX RNA accumulation within the upper leaves. As shown in Figures 1B,C, crops infected with PVX-P0 displayed exacerbations in virulence, and these symptoms were closely correlated with upregulated expression of PVX RNAs. The quantification of RNA accumulation of PVX and the Western blotting analysis further indicated that P0 protein accumulation was significantly higher than that obtained with the PVX vector (Figure 1D). These results suggested that PeVYV P0 has major pathogenicity influences.

### F-Box Motif Is the Necrosis-Inducing Domain of PeVYV P0

**FIGURE 2:**
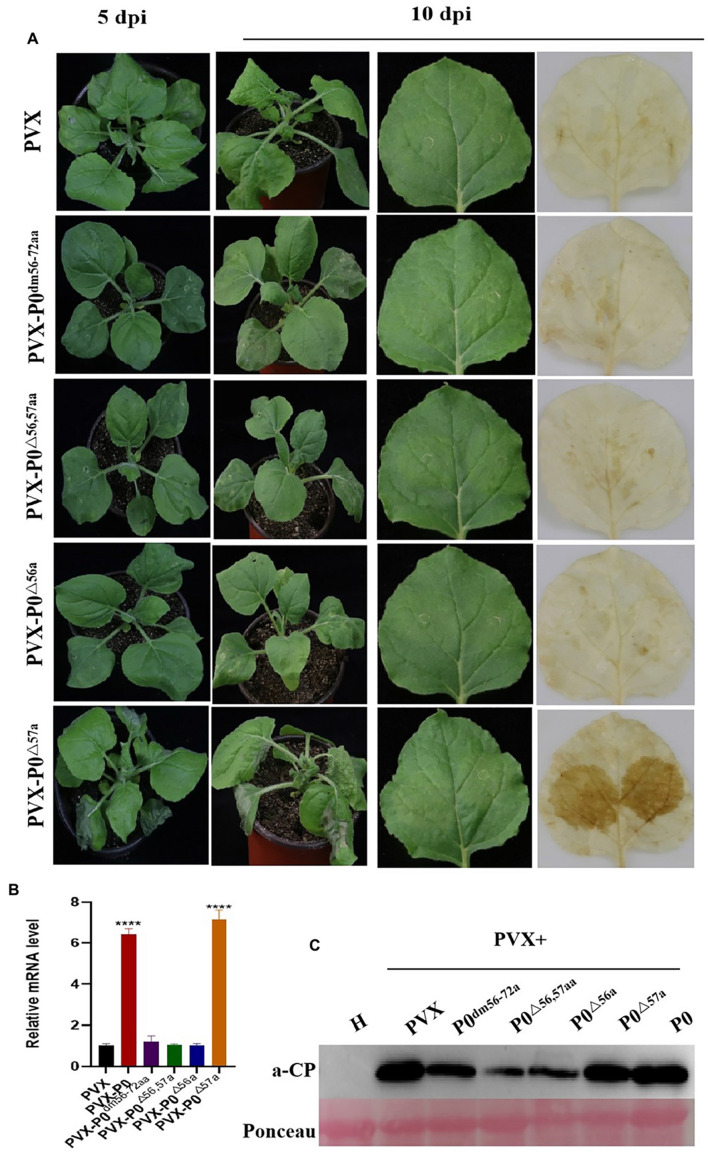
F-box motif of P0 is linked to roles in symptom development. **(A)** Symptom analysis and DAB staining for detecting H_2_O_2_ accumulations in *N. benthamiana* infected with PVX, PVX-P0^*dm56–72aa*^, PVX-P0^△^^56,57aa^, PVX-P0^△^^56a^, and PVX-P0^△^^57a^ variants with mutations in F-box sites. Symptoms were imaged at 5 and 10 dpi. Images shown are representative of six plants infected with each construct in three separate investigations. PVX-P0^△^^57a^-infected plants exhibited a PVX-P0-like phenotype with aggravated necrotic symptoms, PVX-P0^*dm56–72aa*^, PVX-P0^△^^56,57aa^, and PVX-P0^△^^56a^-infected plants showed PVX-like symptoms. Analysis of PVX-, PVX-P0^*dm56–72aa*^-, PVX-P0^△^^56,57aa^-, PVX-P0^△^^56a^-, and PVX-P0^△^^57a^-inoculated *N. benthamiana* plants at 3 dpi revealed that PVX-P0^△^^57a^-afflicted crops exhibited highly concentrated levels of H_2_O_2_ within infected foliage. **(B)** qRT-PCR analysis of PVX CP RNA accumulation in inoculated leaves at 5 dpi. Results showed a higher accumulation of PVX CP RNA in PVX-P0- and PVX-P0^△^^57a^-inoculated leaves. Values are means ± SDs. Highly significant differences (****ρ < 0.0001) between samples in each pair are indicated. **(C)** Western blotting results of PVX CP at 7 to 10 dpi.

All inoculated plants were kept under constant conditions (25°C day/22°C night, 16-h light/8-h dark), and any symptoms were regularly recorded. Six plants were infected by each construct in a minimum of three independent experimental runs. At 5 days post-inoculation (dpi), mild mosaic symptoms were visible on the negative control PVX-infected plants. Similarly, PVX-P0^△^^57a^ caused more rapid, severe, visible necrotic symptoms in the infected foliage at 5 dpi, followed by necrotic lesions throughout the entire plant at 10 dpi (Figure 2A), whereas PVX-P0^*dm56–72aa*^-, PVX-P0^△^^56,57aa^-, and PVX-P0^△^^56*a*^-infected plants demonstrated only mild mosaic symptoms, which was not dissimilar to the findings obtained with plants infected with PVX alone.

Figure 2A also revealed that the H_2_O_2_ content was exacerbated within systemic PVX-P0^△^^57a^-infected leaves at 10 dpi. Conversely, no visible H_2_O_2_ content was identified within PVX-P0^*dm56–72aa*^, PVX-P0^△^^56,57aa^, and PVX-P0^△^^56a^-inoculated foliage.

Fluorescence-based RT-qPCR methodologies were used to quantify PVX RNA within the upper leaves at 5 dpi. As illustrated in Figure 2B, exacerbated virulence was closely correlated with upregulated expression of the PVX transcript. PVX-P0- or PVX-P0^△^^57a^-infected plants exhibited upregulated expression of viral RNA compared with plants infected with PVX-P0^*dm56–72aa*^, PVX-P0^△^^56,57aa^, or PVX-P0^△^^56a^ within the upper leaf foliage.

Western blotting techniques were also adopted to quantify the viral RNA levels, and the results highlighted that PVX, PVX-P0, and PVX-P0^△^^57a^ attained increased viral RNA accumulation within the inoculated leaves at 5 dpi (Figure 2C). In essence, such data and consequent results provide evidence showing that AA 57 of the PeVYV-P0 protein leads to exacerbated symptom development and together aggravates the proliferative rate of PVX to higher levels.

### PeVYV P0 Inhibits Local (Though Not Systemic) Gene Silencing at the Transcriptional Level

**FIGURE 3:**
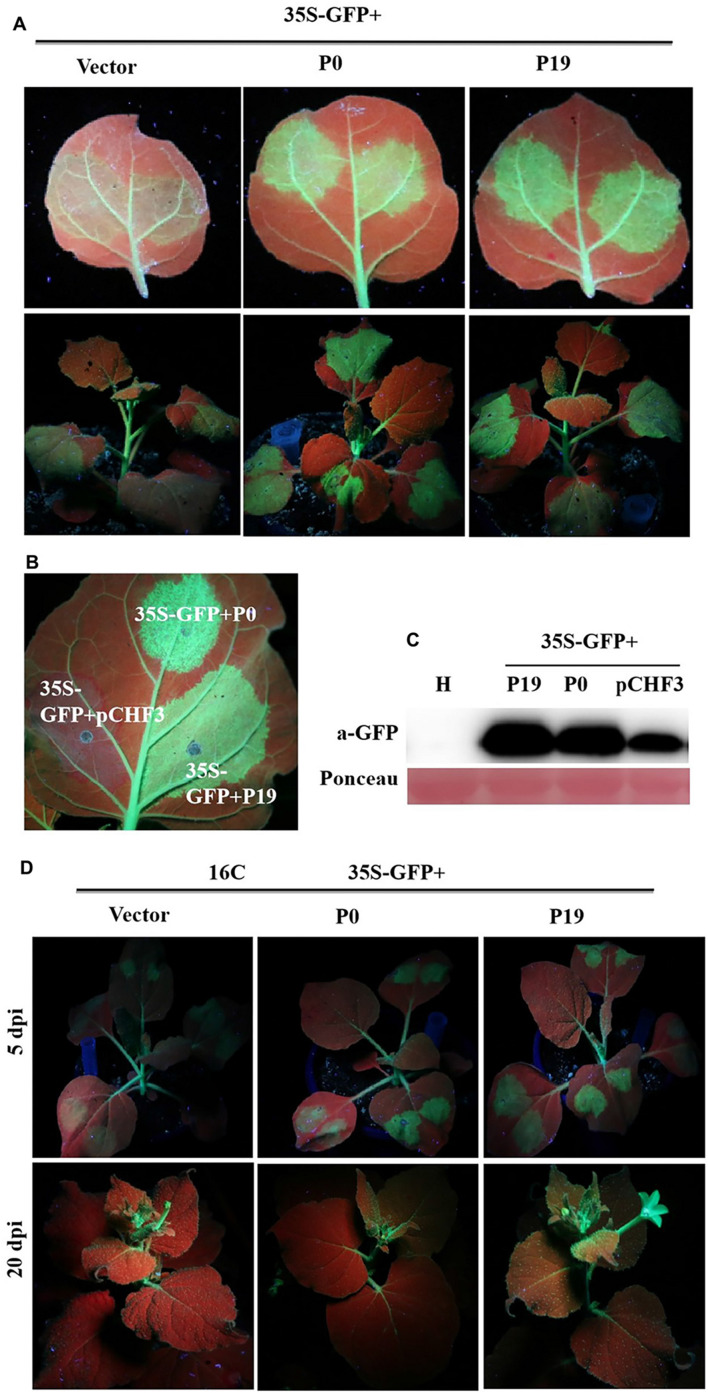
Effect of PeVYV P0 on GFP local/leaf silencing and systemic/whole plant silencing. **(A)** *N*. *benthamiana* 16c plants infected with Agrobacterium culture solutions carrying 35S-GFP and pCHF3 vectors expressing ORF 0 of PeVYV. Images were obtained under UV light with a yellow filter-mounted camera at 5 dpi. **(B)** Inhibition of local/leaf post-translational gene silencing (PTGS) in wild-type *N. benthamiana* crops. *N. benthamiana* crops were infected with Agrobacterium culture solutions as indicated. Images were collected under UV light at 5 dpi. **(C)** Western blotting analysis of GFP within infected *N. benthamiana* leaf patches utilizing a GFP monoclonal antibody. Total soluble protein content collected from *N. benthamiana* crops was needed for recognizing specificity of GFP antibody. Ponceau staining for Rubisco protein (large subunit) served as a loading control. **(D)** PeVYV P0 NA silencing signal transduction within *N. benthamiana* 16c crops. Upper images illustrate *N. benthamiana* 16c crops infected with Agrobacterium cultures bearing 35S-GFP plus empty vector (systemic silencing), 35S-GFP plus PeVYV P0 (systemic silencing), or 35S-GFP plus P19 (no systemic silencing). Images collected using UV light at 20 dpi.

Recent findings demonstrate that P0 proteins of multiple *Polerovirus* species are effective RSSs of PTGS through either local or systemic inhibition of such RNA regulatory processes. To confirm whether P0 can truly inhibit PTGS, concomitant infiltration assays were developed for GFP-transgenic *N. benthamiana* 16c plants, as described earlier. At 5 dpi, P0 + GFP-expressing leaves demonstrated elevated, visible green fluorescence under UV light, which was closely similar to the fluorescent effects exhibited by P19 + GFP-expressing leaves (Figure 3A). This finding was closely correlated with the upregulated expression of GFP, as revealed by Western blotting analyses. However, 35S-GFP + P0/35S-GFP + P19-inoculated foliage exhibited more intense GFP-based fluorescence (Figure 3B). The results from Western blotting analyses were also closely correlated with the GFP fluorescence findings (Figure 3C). In addition, infected crops were scrutinized for possible systemic/whole-plant silencing activities in the upper foliage (i.e., young) at 20 dpi (Figure 3D). The majority of veins within the youngest (uppermost) leaves of the 35S-GFP + P0-infected plants exhibited red coloration under UV illumination, suggesting the onset of RNA silencing mechanisms. Conversely, the GFP fluorescence levels were maintained within the majority of 16c plant foliage leaves inoculated with 35S-GFP + P19. Taken together, the results verify that PeVYV P0 is a powerful local gene silencing suppressor, although it was unable to achieve systemic silencing.

### F-Box Motif Is Needed for PeVYV P0-Induced Post-translational Gene Silencing Inhibition

**FIGURE 4:**
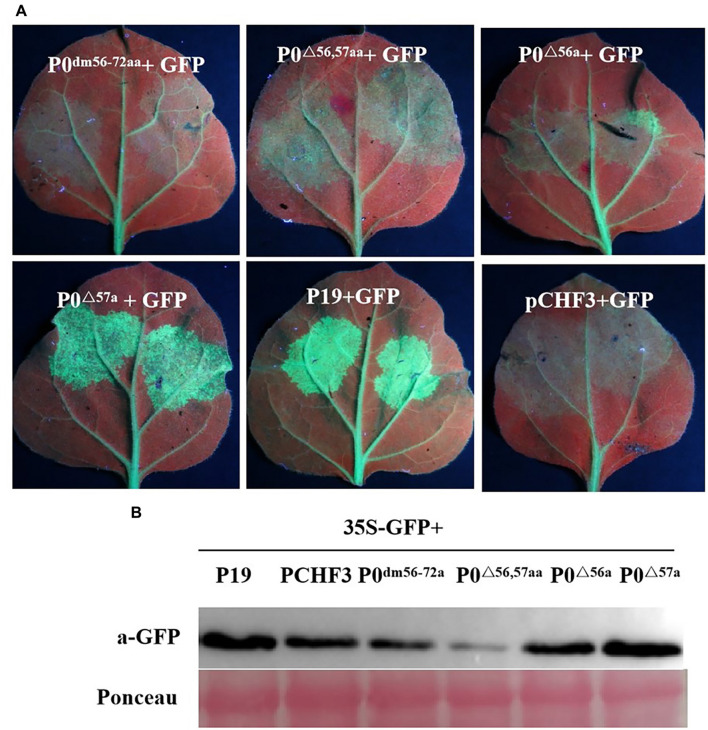
Basic motif deletion in PeVYV P0 affects RNA silencing capacity. **(A)** RSS activity within *N. benthamiana* plants. P0^*dm56–72aa*^, P0^△^^56,57aa^, P0^△^^56a^, and P0^△^^57a^ were infiltrated with *Agrobacterium* cultures carrying specific constructs. Images were obtained under UV light at 5 days post-intervention (dpi). Three separate infiltration assays were performed, and four biological replicates (plants) were included in each assay. **(B)** Western blotting analyses of GFP expression within infiltrated foliage utilizing GFP-specific monoclonal antibodies. Ponceau staining for Rubisco (large subunit) served as a loading control.

Previous studies have suggested that multiple VSR F-boxes are pivotal for RSS activities, and these findings spark the need to confirm such findings, as described earlier. UV-light-monitored leaves demonstrated that the coexpression of 35S-GFP with empty vector, 35S-GFP and P0^*dm56–72aa*^, P0^△^^56,57aa^, or P0^△^^56a^ led to a lack of GFP-based fluorescence at 5 dpi, whereas 35S-GFP + P19 or 35S-GFP + P0^△^^57a^ resulted in intense green fluorescence (Figure 4A). This finding was corroborated through analyses of the relative GFP levels in corresponding leaf patches (Figure 4B), which indicated that the 56LPFLLSSQCPFLSGNTP72 motif and AA 56 of PeVYV are needed by PeVYV P0 to successfully inhibit RNA silencing. These results suggested that AA 56 of the F-box motif is essential for P0 protein function in RNA silencing suppression.

### F-Box Motif Changes the Subcellular Localization of PeVYV P0

**FIGURE 5:**
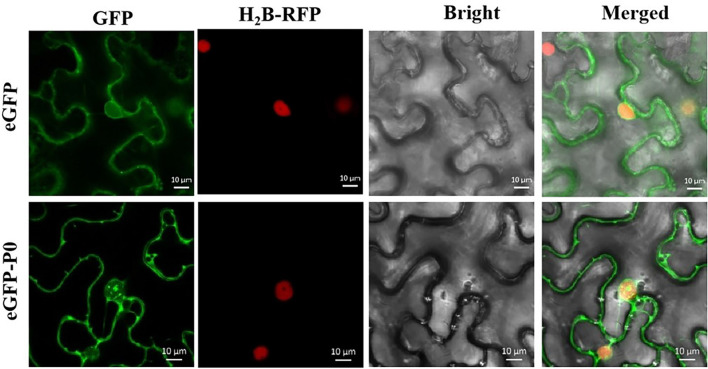
Subcellular localization of PeVYV P0 within *Nicotiana benthamiana* foliage. All pCHF3-eGFP-derived GFP expression-linked fluorescence was dissipated uniformly within cytoplasm/nucleus. P0-GFP fusion protein derived from pCHF3-eGFP is localized within nucleus, particularly within nucleolar regions of virus-infiltrated leaves of *N. benthamiana*. All images were visualized with a confocal microscope (Zeiss LSM880) at 36- to 48-h post-infiltration. Independent infiltration experiments were conducted three times on three separate occasions, and five to six cells were assessed during each investigation.

The development of tools for analyzing subcellular localization and thereby tracking virus-derived proteins was a major step toward unraveling potential roles throughout the course of viral infection. Figure 5 highlights the results from the assays of subcellular localizations within the plants conducted in this study. As predicted, free GFP was identified within the cell membrane and nucleus in a quasi-uniform distribution. The P0-eGFP fusion was found to accumulate within the cell membrane and nucleus, particularly within the nucleolus.

**FIGURE 6:**
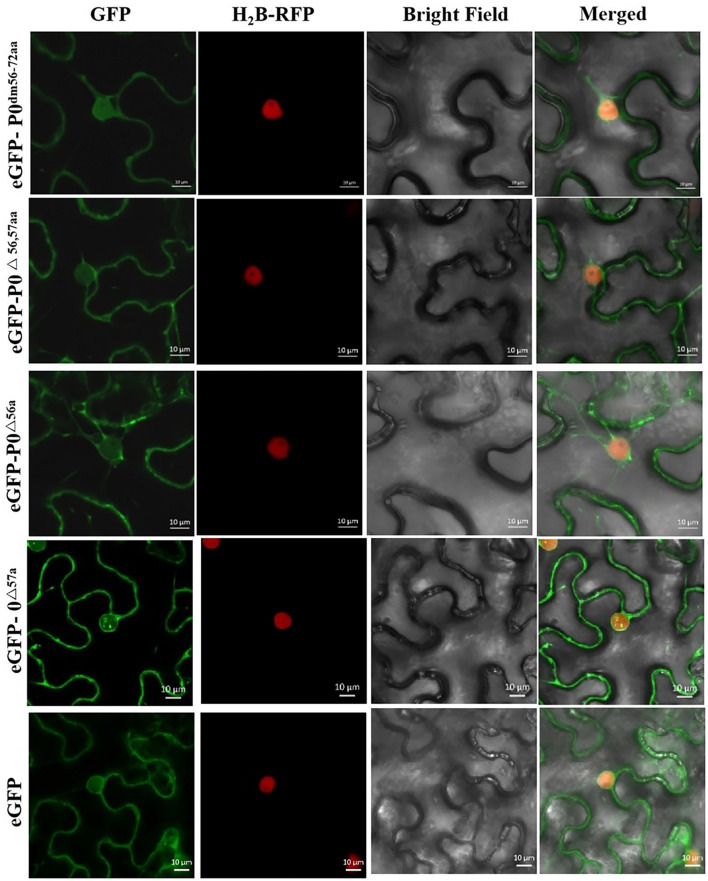
PeVYV P0 motif deletion results in a lack of nucleolar localization. Subcellular localizations of PeVYV P0 and P0 mutants in *N. benthamiana* epidermal cells. Agrobacterium cells carrying P0^*dm56–72aa*^-eGFP, P0^△^^56,57aa^-eGFP, P0^△^^56a^-eGFP, and P0^△^^57a^-eGFP infiltrated *N*. *benthamiana* plant foliage.

Figure 6 shows that the P0^△^^57a^-eGFP fusion aggregated intensely within the nucleus, particularly within the nucleolus. P0^*dm56–72aa*^-eGFP, P0^△^^56,57aa^-eGFP, and P0^△^^56a^-eGFP were found to accumulate in the cell membrane and nucleus, without any nucleolar presence. In essence, the results indicated that the 56LPFLLSSQCPFLSGNTP72 motif and AA 56 of PeVYV P0 are essential for P0 subnuclear localization.

## Discussion

*Polerovirus*-derived P0 proteins are highly effective RSSs in a wide spectrum of viruses, including TuYV, *cucurbit aphid-borne yellows virus* (CABYV), *potato leaf roll virus* (PLRV), *cereal yellow dwarf virus* (CYDV), *beet western yellows virus* (BWYV), *cucurbit aphid-borne yellows virus* (CABYV), *cotton leaf roll dwarf virus* (CLRDV), *melon aphid-borne yellow virus* (MABYV), *beet mild yellowing virus* (BMYV), *maize yellow dwarf virus*-RMV2 (MYDV-RMV2), and *brassica yellows virus* (BrYV) (Pazhouhandeh et al., 2006; Han et al., 2010; Almasi et al., 2015; Cascardo et al., 2015; Chen et al., 2016; Wang et al., 2018; Li et al., 2019). Concomitant-acting roles are typically presented by RSSs, and these include the fulfillment of other non-silencing suppression functions throughout the infective course. Consequently, VSRs have the ability to modulate heterologous system pathogenicity in differing manners (Sharma et al., 2010). TuYV P0^*Tu*^, *potato leafroll virus* P0^*PL*^, and CABYV P0^*CA*^ induce hypersensitive responses in *N*. *glutinosa* accession TW59 (Wang et al., 2015). Conforming the findings from previous investigations, PVX-derived PeVYV P0 expression induced leaf curl manifestations and necrotic presentations that were completely unique compared with the typical symptoms observed in response to PVX infections, whereas mutagenesis of the F-box motif and AA 57, which are both found within the P0 protein, did not lead to necrosis within the afflicted plants. This finding indicated that the F-box-like domain of PeVYV P0 is key in such pathogenic processes, whereas AA 57 plays similar roles in pathogenesis (albeit to a lower extent) and in conferring virulence properties.

The F-box-like domain (N-terminal) of P0 has been identified to play pivotal roles in RSS activities (Han et al., 2010; Fusaro et al., 2012). There are two conserved domains in the P0 protein: the consensus F-box-like motif (LPXX(L/I)X10–13P) and the Phe/Trp (FW) remnants at the C-terminal consensus sequence [(K/R) IYGEDGX3FWR] (Han et al., 2010; Fusaro et al., 2012; Almasi et al., 2015; Chen et al., 2016). MYDV-RMV2 P0 functions such as a typical F-box-like motif, and mutagenesis of Ala [positions 67, 68, and 81 in the F-box-like motif (67LPxx81P)] fully inhibits P0-induced RSS activities (Wang et al., 2018). The F-box-like domain is essential for SSP functional roles, although the LP motif is redundant for such activities. The introduction of a 3× AA substitution event within *pea enation mosaic virus-1* (PEMV-1) P0 (leucine-124, proline-125, and proline-133 converted to alanine residues) produced a protein (P0PEΔLPP) without RSS activities, which is actually the opposite of our previous findings (Fusaro et al., 2012).

Trp 212 is a key player in the RSS mechanism within the MABYV P0 protein (Han et al., 2010). Our findings are similar to those found for the P0 protein of *Polerovirus* by previous authors. Our results show that the F-box-like domain and AA 56 of P0 are essential for green fluorescence in *N. benthamiana* 16c. In addition, PeVYV P0 inhibits local, although not systemic, RNA silencing mechanisms. This finding is similar to those found with TuYV, CABYV, PLRV (EU variant), MABYV, and BMYV P0 proteins, all of which are SSPs only at the local RNA silencing level (Han et al., 2010). However, the P0 protein found in other viruses, such as *sugarcane yellow leaf virus* (SCYLV) and PEMV-1 (genus *Enamovirus*), manages to perform RSS functions at the local and systemic levels (Li et al., 2019), which highlights that the P0 proteins from multiple viruses derived from *Poleroviruses* display different RSS capacities.

The nature of the subcellular localizations leads to a better understanding of viral interactions with key host molecular players and the viral roles during the course of infection. Similar to poleroviral P0 proteins, PEMV-1 P0 is located in the nucleus (Fusaro et al., 2012). MYDV-RMV2 P0 has been found in both the nucleus and cytoplasm (Wang et al., 2018). This study revealed that the PeVYV P0 protein tended to exhibit a complex localization, with an intense presence within the cytoplasm, nucleus, and nucleolus. The mutagenesis of AA 56 on P0 led to the dissipation of the protein from the cytoplasm/nucleus, indicating that the mutated variant of this protein was localized in the nucleolus.

Point mutations within the P0 F-box motif could inhibit SKP1 interactions, consequently lowering AGO1 protein destabilizing events and eventually leading to viral pathogenicity (Derrien et al., 2012, 2018; Li et al., 2019). The TuYV, CABYV, and BrYV P0 proteins contain an F-box-like domain that interacts with SKP1 (Li et al., 2019). BrYV-A P0 BrA also interacts with NbRAF2, which consequently alters the localization distribution profile to enhance viral invasion (Sun et al., 2018). However, P0 fails to help proteasome-directed degradation events, as predicted, and has rather been found to recognize AGO1-derived DUF1785 domain degradation and consequently initiate autophagy-mediated AGO1 (Derrien et al., 2012, 2018). Most likely, the P0 protein encoded by PeVYV interacts with related genes in the ubiquitination or AGO pathways or other verified host factors, and the results scientifically uncover the novel molecular mechanisms underlying PeVYV–host interactions.

## Conclusion

Our results validated that the P0 protein encoded by the initial ORF of PeVYV P0 has RSS activity, induces hypersensitivity responses that regulate viral propagation, and leads to apoptosis. All these activities tend to stem from AA 56 or AA 57 of the P0-derived F-box-like domain. The outcomes obtained here suggest that further studies should be conducted on the molecular mechanisms of amino acids of the F-box domain of P0 protein in the interaction of PeVYV with plants.

## Data Availability Statement

The original contributions presented in the study are included in the article/supplementary material, further inquiries can be directed to the corresponding author.

## Author Contributions

LW, PT, and CL carried the experimental work. SZ and XHY collected and analyzed the data. XLY, XZ, and YL designed study, guided data interpretation, and wrote the manuscript. All authors approved the manuscript before it was submitted by the corresponding author.

## Conflict of Interest

The authors declare that the research was conducted in the absence of any commercial or financial relationships that could be construed as a potential conflict of interest.

## Publisher’s Note

All claims expressed in this article are solely those of the authors and do not necessarily represent those of their affiliated organizations, or those of the publisher, the editors and the reviewers. Any product that may be evaluated in this article, or claim that may be made by its manufacturer, is not guaranteed or endorsed by the publisher.
